# Cilostazol Attenuates Ovariectomy-Induced Bone Loss by Inhibiting Osteoclastogenesis

**DOI:** 10.1371/journal.pone.0124869

**Published:** 2015-05-18

**Authors:** Ke Ke, Ali Muhammad Safder, Ok-Joo Sul, Jae-Hee Suh, Yeonsoo Joe, Hun-Taeg Chung, Hye-Seon Choi

**Affiliations:** 1 Department of Biological Sciences, University of Ulsan, Ulsan 680–749, Korea; 2 Department of Pathology, Ulsan University Hospital, Ulsan 682–714, Korea; Faculté de médecine de Nantes, FRANCE

## Abstract

**Background:**

Cilostazol has been reported to alleviate the metabolic syndrome induced by increased intracellular adenosine 3’,5’-cyclic monophosphate (cAMP) levels, which is also associated with osteoclast (OC) differentiation. We hypothesized that bone loss might be attenuated via an action on OC by cilostazol.

**Methodology and Principal Findings:**

To test this idea, we investigated the effect of cilostazol on ovariectomy (OVX)-induced bone loss in mice and on OC differentiation in vitro, using μCT and tartrate-resistant acid phosphatase staining, respectively. Cilostazol prevented from OVX-induced bone loss and decreased oxidative stress in vivo. It also decreased the number and activity of OC in vitro. The effect of cilostazol on reactive oxygen species (ROS) occurred via protein kinase A (PKA) and cAMP-regulated guanine nucleotide exchange factor 1, two major effectors of cAMP. Knockdown of NADPH oxidase using siRNA of p47^phox^ attenuated the inhibitory effect of cilostazol on OC formation, suggesting that decreased OC formation by cilostazol was partly due to impaired ROS generation. Cilostazol enhanced phosphorylation of nuclear factor of activated T cells, cytoplasmic 1 (NFAT2) at PKA phosphorylation sites, preventing its nuclear translocation to result in reduced receptor activator of nuclear factor-κB ligand-induced NFAT2 expression and decreased binding of nuclear factor-κB-DNA, finally leading to reduced levels of two transcription factors required for OC differentiation.

**Conclusions/Significance:**

Our data highlight the therapeutic potential of cilostazol for attenuating bone loss and oxidative stress caused by loss of ovarian function.

## Introduction

Postmenopausal osteoporosis is a prominent symptom of loss of ovarian function, which is characterized by decreased estrogen and increased follicle-stimulating hormone (FSH). For decades bone loss was solely attributed to the declining estrogen levels [1], but the sharply increased FSH levels also contribute to the bone loss [2], implying a role of FSH in controlling bone mass. FSH enhances the differentiation and function of osteoclasts (OC) via lowering adenosine 3’,5’-cyclic monophosphate (cAMP) in OC [2]. The net intracellular level of cAMP is modulated by adenylate cyclase and cyclic AMP-dependent phosphodiesterases (PDE). PDE inhibitors enhance bone mass in mice [3, 4], implying that cAMP might play a role in maintaining bone mass.

Cilostazol [6-[4-(1-cyclohexyl-1H-tetrazol-5-yl)butoxyl]-3,4- dihydro-2 (1H)-quinolinone], an inhibitor of PDE3, has been used as a vasodilating agent to treat arterial obstruction [5]. Recent studies have broadened its application as it has anti-inflammatory [6] and antiatherogenic actions [7]. It attenuates atherosclerosis by inhibiting NF-κB activation and so reducing levels of superoxide and tumor necrosis factor (TNF)-α with a significant elevation of cAMP expression in the mice fed cilostazol-supplemented high fat diet [7]. An ability of cilostazol to scavenge hydroxyl and peroxyl radicals also has been demonstrated in endothelial cells [8]. These studies suggest that cilostazol has an anti-oxidative and anti-inflammatory activity by up-regulating cAMP.

Bone is a dynamic tissue homeostatically controlled by the balance between OC-mediated bone resorption and osteoblast-mediated bone formation. Postmenopausal osteoporosis results from an excess of bone resorption [2, 9]. The augmented resorption could be caused by elevated number of OC due to increased formation and/or survival of OC. OCs are derived from hematopoietic monocyte/macrophage lineage cells and are specialized giant multinucleated cells. They participate in physiological bone remodeling as well as in the bone destruction associated with chronic inflammatory disease [10]. They are under the control of two cytokines produced by bone marrow mesenchymal cells, macrophage-colony stimulating factor (M-CSF) and receptor activator of nuclear factor-κB ligand (RANKL) [11]. RANKL is required for OC function as well as OC differentiation [12]. Cross-linking of RANKL to its receptor, RANK, activates cytoplasmic TNF receptor-associated factor 6 that led to the activation of key transcription factors, nuclear factor-κB (NF-κB) and nuclear factor of activated T cells, cytoplasmic 1 (NFAT2), resulting in enhanced expression of OC-specific genes [13, 14]. Ectopic expression of NFAT2 induces osteoclastogenesis without RANKL, and OC can not be differentiated from NFAT2-deficient embryonic stem cells [13], suggesting a critical role of NFAT2 in osteoclastogenesis.

We hypothesized that cilostazol protects from OVX-induced bone loss by elevating intracellular cAMP. Our present studies demonstrated that cilostazol elevated intracellular cAMP in OC and reduced ROS production.

## Results

### Cilostazol attenuates ovariectomy (OVX)-induced bone loss in mice

**Fig 1:**
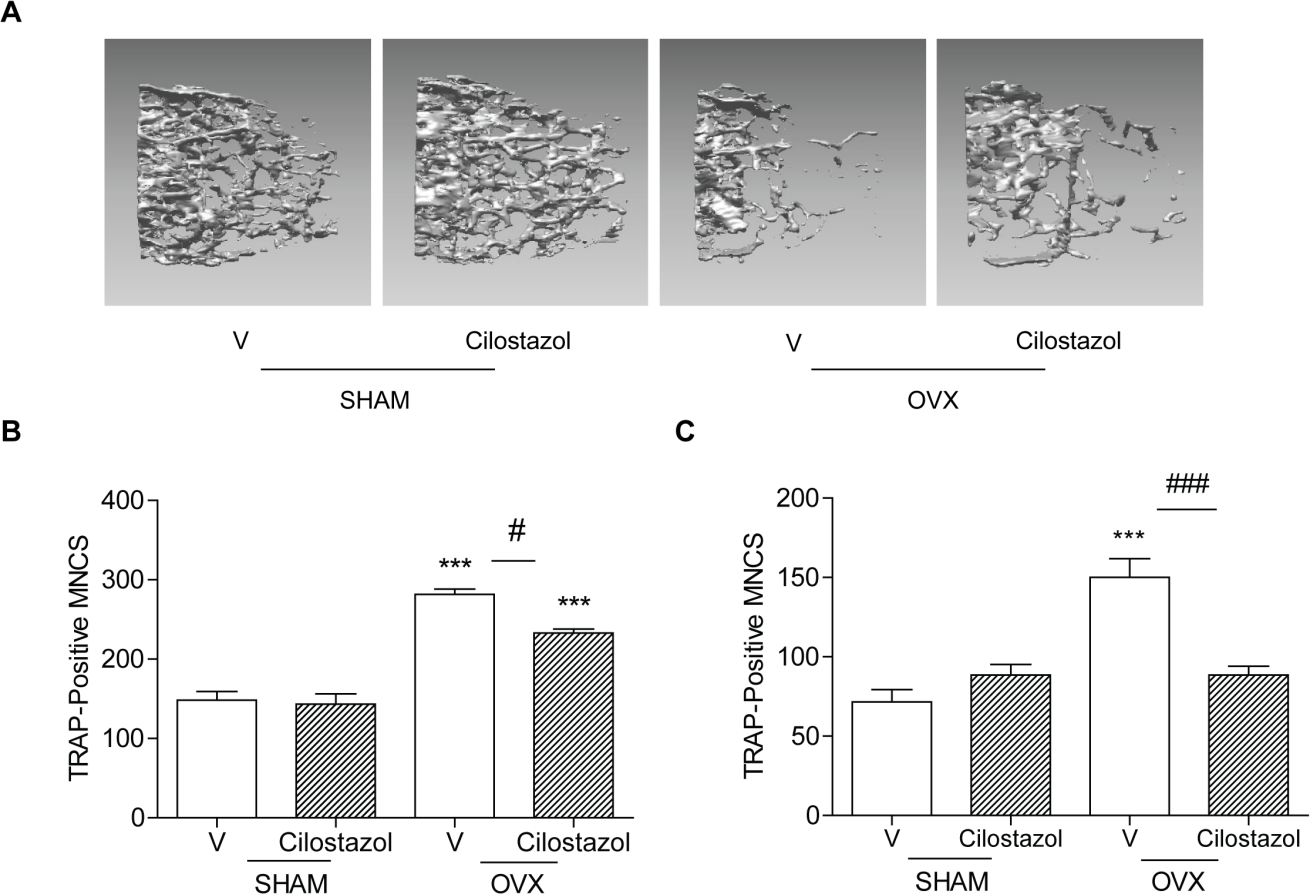
Cilostazol attenuates OVX-induced bone loss in mice. Bone densities of femora were measured on vehicle (V)-treated (SHAM, n = 5; OVX, n = 7), cilostazol (0.5 mg/kg/d)-treated (SHAM, n = 6; OVX, n = 7) mice 8 weeks after surgery. Representative μCT images of distal femora (1.0 mm from the growth plate of the distal femur) (A). Numbers of OCs in cultures of enriched BMM (5 x 10^3^ cells/well) (B) and whole bone marrow (2 x 10^4^ cells/well) (C) stimulated with RANKL/M-CSF and 1,25(OH)_2_D_3_, respectively were counted by an experienced observer who was blinded to each treatment for quantification of TRAP-positive MNC/ each well using an eye piece graticule at a magnification of Χ100. Results were expressed as means ± SEM of 3–6 cultures per variable. ***, *p*<0.001 compared with vehicle-treated SHAM. ^#^, *p*<0.05; ^###^, *p*<0.001 compared with vehicle-treated OVX. Similar results were obtained in three independent experiments.

To evaluate the effect of cilostazol on OVX-induced bone loss, micro CT (μCT) of femurs from OVX mice treated with cilostazol or vehicle was analyzed, and compared with sham surgery. No significant differences in body size and shape were observed between cilostazol and vehicle-treated OVX mice at 14 weeks of age. Treatment of cilostazol attenuated bone loss induced by OVX, but had no significant change on sham mice (**Fig 1A, Table 1**). It increased bone mineral density (BMD), bone volume (BV/TV), and trabecular number (Tb. N.), and reduced the enlargement of trabecular space (Tb. Sp.) compared with OVX alone (**Table 1**). It also reduced the increase of OC induced by OVX (**Table 1**). Consistent with these findings, numbers of OC in ex vivo cultures of BMM-enriched population from cilostazol-treated OVX mice were lower than in those from OVX mice (**Fig 1B**), indicating that in vivo treatment with cilostazol limited the increase in OC resulting from OVX. Consistently, serum collagen-type I fragments (CTX-1), an in vivo bone resorption marker was also reduced when cilostazol was administered to OVX mice (**Table 1**). However, cilostazol did not significantly affect the in vivo bone formation markers, serum alkaline phosphatase (ALP) and osteocalcin (**Table 1**), although both were elevated in OVX mice, indicating that cilostazol may exert bone protection through its action on OC, not on osteoblasts. In agreement with this, ex vivo cultures of whole bone marrow showed a similar trend to those of the enriched bone marrow-derived macrophage (BMM) (**Fig 1C**), consistent with the absence of an effect on osteoblasts. Cilostazol also reduced the rise in serum H_2_O_2_ induced by OVX (**Table 1**), indicating that it opposed the oxidative stress induced by OVX.

**Table 1:** Trabecular microarchitecture and biochemical markers of OVX and SHAM mice treated with cilostazol at 8-weeks after surgery.

	SHAM		OVX	
	V	Cilostazol	V	Cilostazol
BMD [mg/cm^3^]	58.44±1.880	69.00±5.488	35.50±1.659^α’^	53.77±7.667^c^
BV/TV [%]	9.434±0.3900	10.10±0.4980	4.371±0.2670^α”^	6.424±0.5000^c^ ^d”^
Tb. N. [mm^-1^]	1.391±0.0450	1.620±0.0750^b^	0.6670±0.0240^α”^	0.8960±0.0660^c^ ^d”^
Tb. Sp. [mm]	0.3170±0.0050	0.3030±0.0110	0.4770±0.0110^α”^	0.4400±0.0080^c^ ^d”^
CTX-1 [ng/ml]	17.64±1.914	21.08±0.8780	27.04±1.758^α’^	19.90±2.096^c^
OCN [ng/ml]	13.25±1.026	15.53±0.5050	17.32±1.155^α”^	18.51±1.376^d”^
ALP [U/L]	16.93±0.9180	19.00±1.654	25.87±2.568^α’^	23.19±1.501^d^
H_2_O_2_ [μM/ml]	10.11±0.9070	12.47±0.6080	13.62±0.5070^α’^	11.59±0.4260^c^
OC.N/BS [mm^-1^]	2.750±0.1640	3.000±0.3650	6.000±0.3780^α”^	4.333±0.2110^c’^ ^d’^

V-treated (SHAM, n = 5; OVX, n = 7); cilostazol (0.5 mg/kg/d)-treated (SHAM, n = 6; OVX, n = 7). Data are represented as means±SEM. Differences between groups were analyzed by one-way ANOVA, followed by Bonferroni post tests.

^α’^ *p*<0.01

^α”^ *p*<0.001, SHAM/V (vehicle) vs. OVX/V

^b^ *p*<0.05, SHAM/V vs. SHAM/Cilostazol

^c^ *p*<0.05

^c’^, *p*<0.01, OVX/V vs. OVX/Cilostazol

^d^ *p*<0.05

^d’^ *p*<0.01

^d”^ *p*<0.001, SHAM/V vs. OVX/Cilostazol)

### Cilostazol inhibits OC differentiation

**Fig 2:**
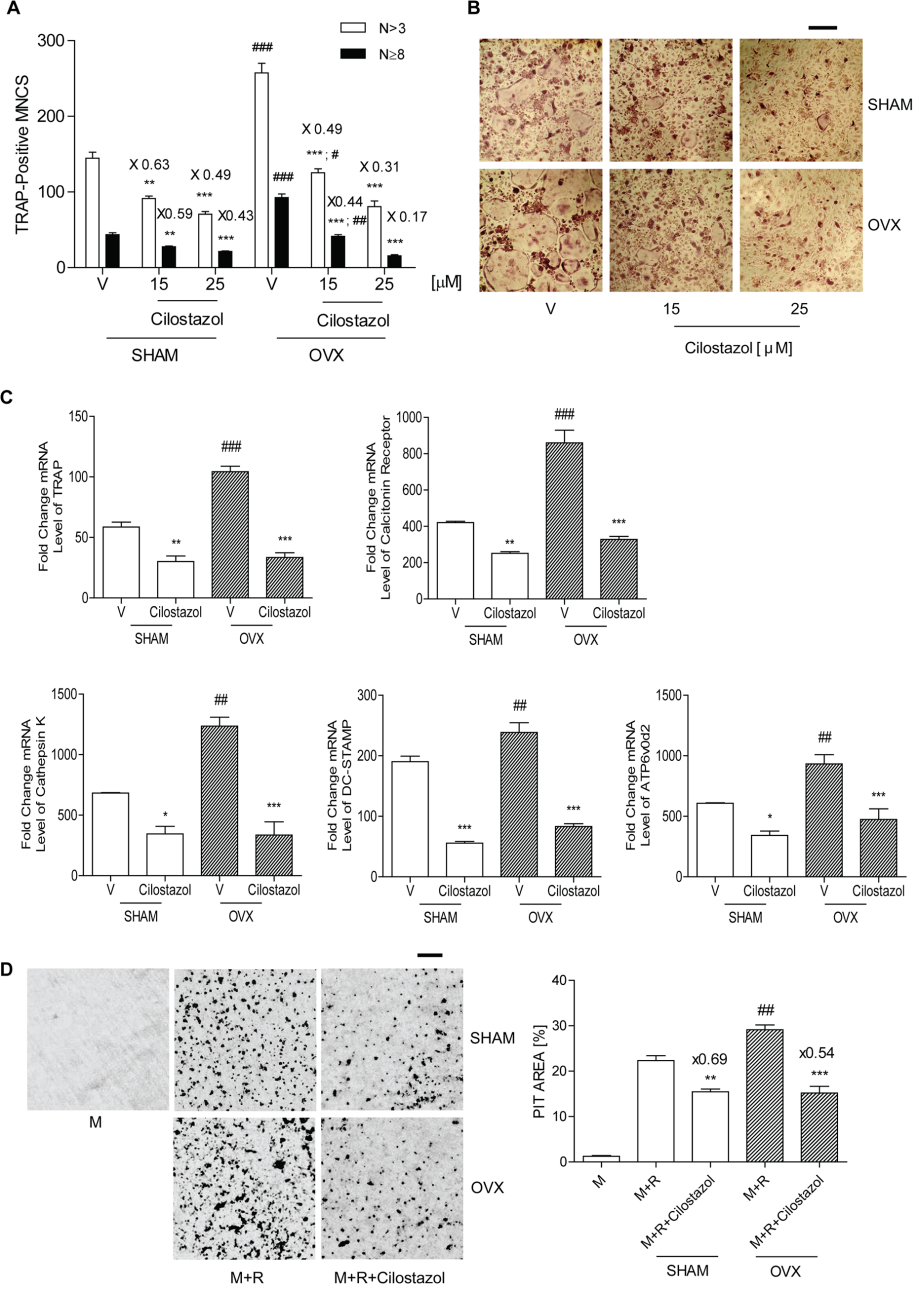
Cilostazol decreases OC formation and bone resorption induced by RANKL. (A) BMM (10^4^ cells/well) from sham and OVX mice were incubated with cilostazol (0, 15, 25 μM) in the presence of M-CSF (20 ng/ml) and RANKL (40 ng/ml). After 3 d, cells were fixed and stained for TRAP. Numbers of OCs were counted by an experienced observer who was blinded to cilostazol dose for quantification of TRAP-positive MNC/each well using an eye piece graticule at a magnification of Χ100. Results were expressed as means ± SEM of 3–6 cultures per variable. Frequency distribution of OCs according to number of nuclei. (B) Representative photos of A. Scale bar, 200 μm. Means of the 3 groups are significantly different (*P* <0.001). **, *P* <0.01; ***, *P* <0.001 compared with vehicle (V)-treated cells in sham and OVX. ^#^, *P* <0.05; ^##^, *P* <0.01; ^###^, *P* <0.001 sham vs. OVX. Numbers above the histogram are ratios of the number of TRAP-positive MNC in the presence of cilostazol to in its absence for each group. (C) BMMs (5 x 10^5^ cells/well) from sham and OVX mice were incubated with cilostazol (25 μM) in the presence of M-CSF and RANKL for 48 h; total RNA was extracted and subjected to qPCR analysis for TRAP, calcitonin receptor, cathepsin K, DC-STAMP, and ATP6v0d2. *, *P*<0.05; **, *P*<0.01; ***, *P* <0.001 compared with V in sham and OVX. ^##^, *P* <0.01; ^###^, *P* <0.001 sham vs. OVX. No significant difference was observed between sham vs. OVX in the presence of cilostazol. (D) RANKL-induced mature OC (~1000 cells) from sham and OVX mice were incubated with or without cilostazol (25 μM) on dentine slices for 24 h, and the slices were stained for pit formation. Representative photos of the resorption pits in V- and cilostazol-treated slices are shown. Scale bar, 50 μm. **, *P*<0.01; ***, *P*<0.001 compared with V in sham and OVX. ^##^, *P* <0.01 sham vs, OVX. Numbers above the histogram are ratios of pit area of in the presence of cilostazol to in its absence for each group. The areas of the resorption pits per dentine slice were quantified blind using the ImageJ 1.37v program. Similar results were obtained in three independent experiments.

We investigated the effects of cilostazol on OC formation in cultures of BMM from sham and OVX mice free of stromal cells and lymphocytes, to assess whether it modulated osteoclastogenesis. Three day exposure to M-CSF and RANKL which are essential for OC differentiation resulted in maximal OC formation. Osteoclastogenesis was inhibited by cilostazol in a concentration-dependent manner as shown by counting TRAP-positive MNC (**Fig 2A and 2B**). The inhibitory effect of cilostazol on OC was more prominent in more highly fused OC (N≥8). Consistent with this result, transcripts of DC-STAMP and ATP6v0d2 were reduced in cilostazol-treated cells (**Fig 2C**). Transcripts of TRAP, calcitonin receptor, and cathepsin K were also reduced after 48 h of RANKL stimulation (**Fig 2C**). Cilostazol did not have any detrimental effect on the viability of BMM under the assayed conditions (**S1 Fig**). Nor did it decrease the number of OC by enhancing apoptosis of mature OC (**S2 Fig**). Next, we examined whether it also alleviated bone resorption. Mature OC formed substantial numbers of pits on dentine slices, and the addition of cilostazol decreased the overall area of pits (**Fig 2D**), suggesting that cilostazol inhibits the activity of OC. The decrease of pit areas by cilostazol was greater in mature OC from OVX mice than that from sham mice.

### Cilostazol increases intracellular cAMP and decreases long-lasting levels of ROS to decrease OC differentiation via protein kinase A and cAMP-regulated guanine nucleotide exchange factor 1

**Fig 3:**
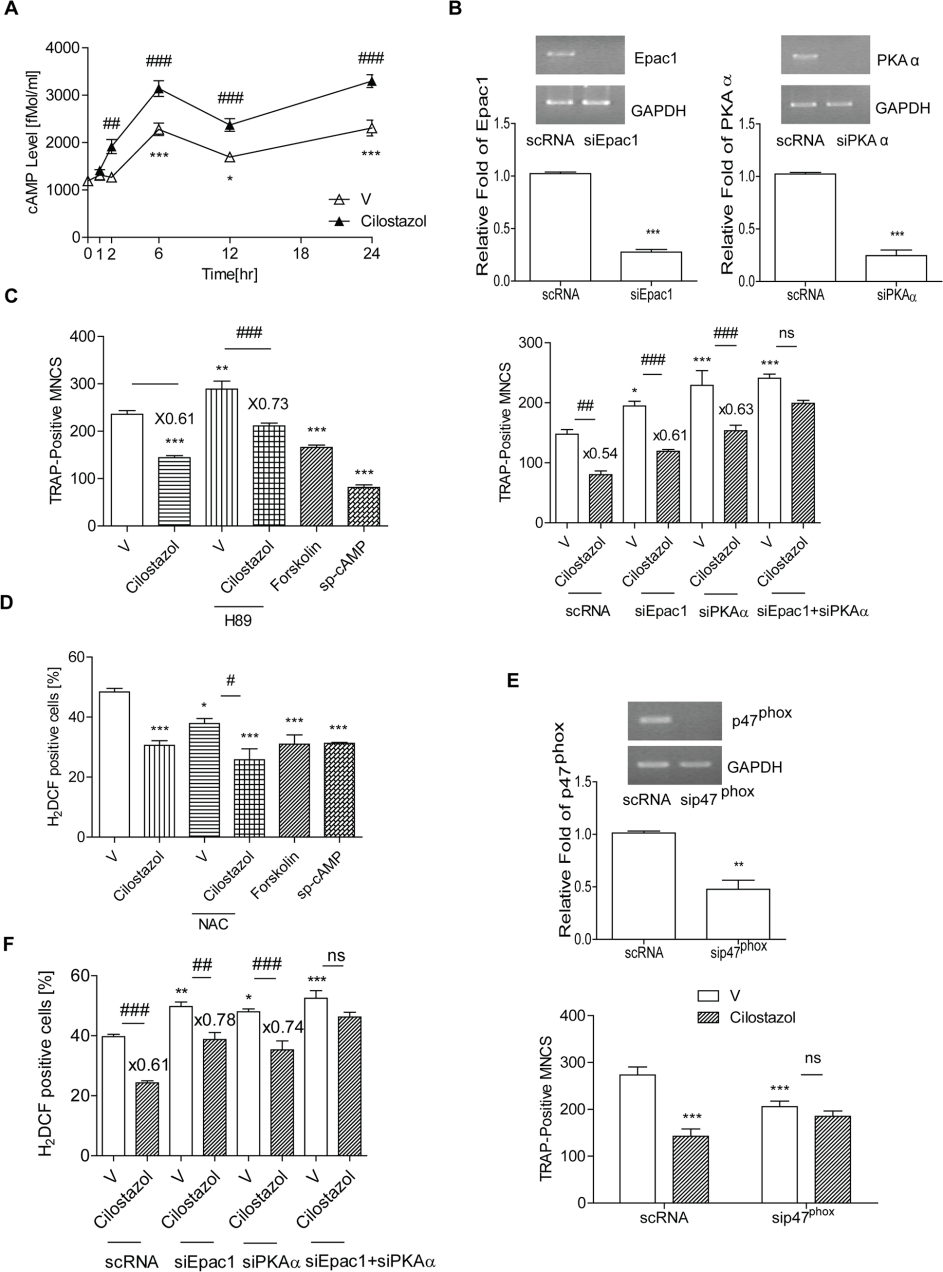
Cilostazol increases intracellular cAMP which reduces ROS and OC formation via PKA and Epac1. (A) Intracellular levels of cAMP upon stimulation with M-CSF and RANKL in the presence or absence of cilostazol (30 μM) for 24 h were determined using an ELISA for cAMP. Intracellular cAMP was increased after 6 h exposure to M-CSF and RANKL. *, *p*<0.05; ***, *p*<0.001 compared with 0 h. ^##^, *p*<0.01; ^###^, *p*<0.001 compared with vehicle (V)-treated cells. (B, F) BMM (2 x 10^4^ cells/well) were transfected with siPKAα, siEpac1 or scRNA. Down-regulation of PKA, Epac1 by siRNA was confirmed by RT-PCR and qPCR. ***, *p*<0.001 compared with scRNA. After transfection with each siRNA, cells were treated with cilostazol and stimulated with RANKL. TRAP-positive MNCs were counted after 72 h. (B) and ROS levels were measured after 48 h (F). *, *p*<0.05; ***, *P*<0.001 compared with V of scRNA-transfected cells. ^##^, *p*<0.01; ^###^, *p*<0.001 compared with V of each siRNA-transfected cells. Numbers above the histograms are ratios of the number of MNC (B) or ROS-positive cells (F) in the presence of cilostazol to in its absence. (C) BMM were stimulated with M-CSF and RANKL along with H-89 (1 μM) in the presence or absence of cilostazol (30 μM), forskolin (1 μM), or sp-cAMP (10 μM) for 3 d and TRAP-positive MNCs were measured). **, *p*<0.01; ***, *P*<0.001 compared with vehicle. ^###^, *p*<0.001 compared with V of H-89-treated cells. Numbers above the histogram are ratios of the number of TRAP-positive MNC in the presence of cilostazol to in its absence for each group. (D) Intracellular levels of ROS upon stimulation with RANKL in the presence or absence of cilostazol (30 μM) for 48 h were determined using H_2_DCFDA. ROS levels were quantified by flow cytometry. (E) BMMs were transfected with sip47^phox^ or scRNA. Down-regulation of p47^phox^ by siRNA was confirmed by RT-PCR and qPCR. **, *p*<0.01 compared with scRNA. After transfection with siRNA, cells were treated with cilostazol and stimulated with RANKL for 72 h and TRAP-positive MNCs counted. ***, *P*<0.001 compared with V of scRNA-transfected cells. There was no significant difference between V and cilostazol after transfection of sip47^phox^. Numbers of OCs were counted blind by quantification of TRAP-positive MNC/ each well using an eyepiece graticule at a magnification of Χ100. Results were expressed as means ± SEM of 3–6 cultures per variable. Similar results were obtained in three independent experiments.

A hint that cAMP may affect osteoclastogenesis was suggested by the finding that FSH increased osteoclastogenesis by decreasing intracellular cAMP [2], while forskolin inhibited osteoclastogenesis [15]. We found that cilostazol, a PDE3 inhibitor, increased intracellular cAMP during osteoclastogenesis. As shown in **Fig 3A**, intracellular cAMP was increased after 6 h exposure of BMM to RANKL and M-CSF. Intracellular cAMP upon cilostazol treatment started to increase by 2 h, reached a maximum at 6 h, and stayed up for 24 h.

Intracellular cAMP signaling is mediated by two types of effector that bind cAMP, namely protein kinase A (PKA) and cAMP-regulated guanine nucleotide exchange factors (cAMP-GEF, Epac1 and Epac2) [16]. To examine which were involved in the inhibitory effect of cilostazol during osteoclastogenesis, we treated cells with siRNA specific for either the PKA catalytic subunit α (PKAα) or Epac1, which is more widely expressed than Epac2, and then exposed them to cilostazol. As shown in **Fig 3B**, knock-down of either PKA or Epac1 increased TRAP-positive MNCs, indicating that cAMP-mediated signaling acts as a negative modulator of osteoclastogenesis. Consistent with this, a cell-permeable PDE-resistant cAMP analogue (sp-cAMP) or forskolin alone decreased OC formation (**Fig 3C**). The inhibitory effect of cilostazol on OC formation was partially inhibited in the presence of siPKAα or siEpac1 separately. However, co-transfection of siPKAα and siEpac1 resulted in complete inhibition (**Fig 3B**), indicating that the inhibitory effect of cilostazol on OC formation requires collaboration between PKA and Epac1.

Since *in vivo* administration of cilostazol reduced the increase of serum ROS due to OVX, we examined whether it also decreased ROS in OC. Osteoclastogenic cytokine (RANKL and M-CSF)-stimulated ROS reached a maximum after 48 h of exposure and decreased upon treatment with the ROS scavenger, N-acetyl cysteine (NAC); cilostazol decreased ROS in the OC (**Fig 3D**), as did forskolin and sp-cAMP (**Fig 3D**), suggesting that cAMP is responsible for decreasing ROS in OC. Cilostazol and NAC acted additively to reduce ROS (**Fig 3D**), implying that the effect of cilostazol on ROS may be caused by impaired ROS generation rather than ROS scavenging. Knockdown of p47^phox^ by siRNA decreased OC formation and abolished the inhibitory effect of cilostazol on OC formation (**Fig 3E**). Co-transfection of siPKA and siEpac1 also abolished the inhibitory effect of cilostazol on ROS level, while each alone had a partial effect (**Fig 3F**), indicating that both PKA and Epac1 are necessary for cilostazol to decrease ROS.

### Cilostazol impairs activation of two key transcription factors for osteoclastogenesis, NF-κB and NFAT2

**Fig 4:**
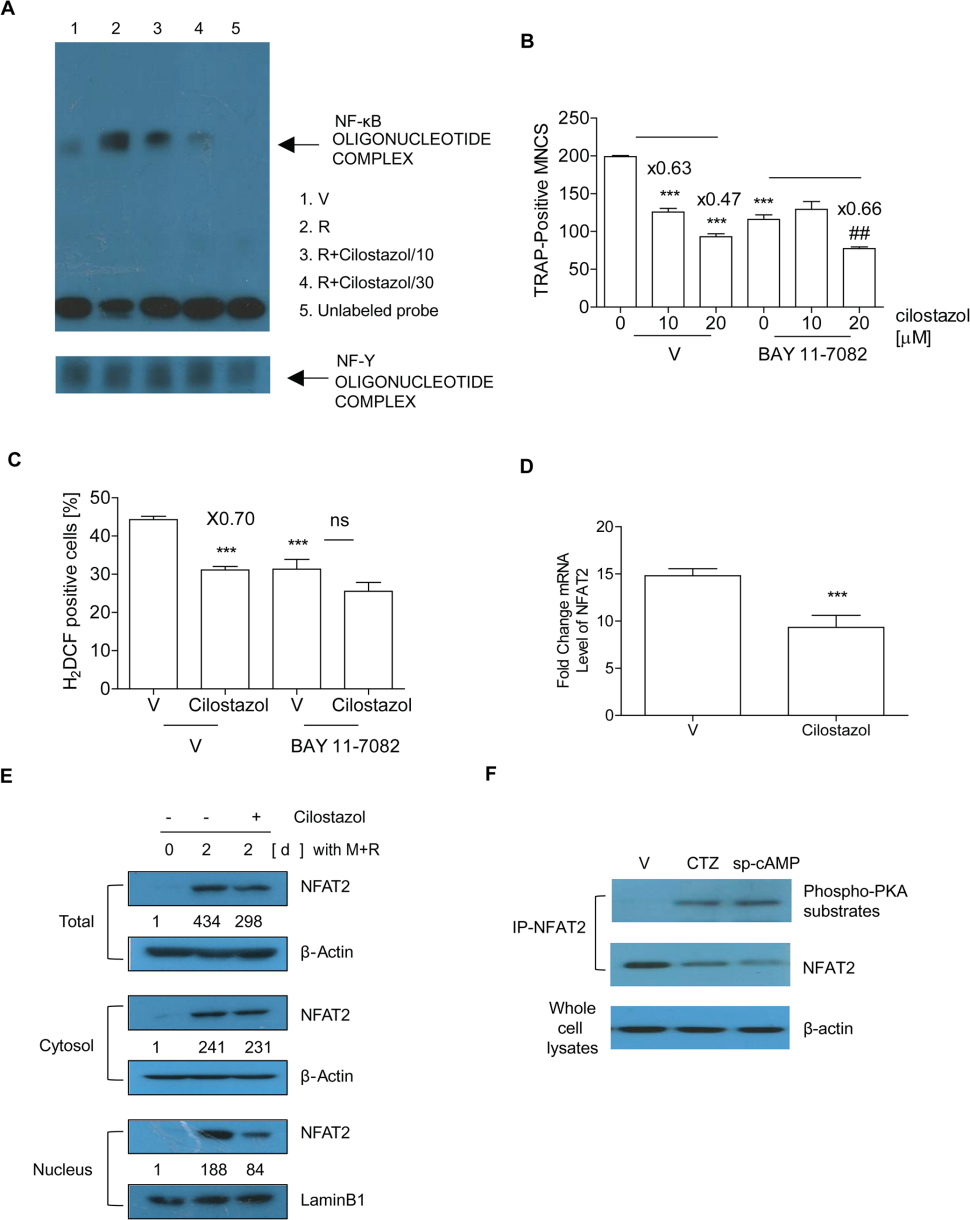
Cilostazol impairs activation of two key transcription factors for osteoclastogenesis, NF-κB and NFAT2. (A) BMM (5 x 10^6^ cells/plate) were stimulated with vehicle (V) (lane 1) or RANKL (lane 2) along with cilostazol (10 μM, lane 3; 30 μM, lane 4) for 1 h. Hundred-fold excess of unlabeled probe (lane 5) was used as a negative control. NF-Y DNA binding activity was measured as an internal control. (B-D) BMM with cilostazol (30 μM) in the presence or absence of BAY 11–7082 (1 μM) were incubated with M-CSF and RANKL for 72 h to count TRAP-positive MNCs (B) and for 48 h to determine ROS level (C) and extract RNA (D). Numbers above the histograms are ratios of the number of MNC (B) or ROS-positive cells (C) in the presence of cilostazol to in its absence. Total RNA was extracted and subjected to qPCR analysis for NFAT2. The expression level before RANKL treatment was set at 1 (D). **, *P*<0.01, ***, *P*<0.001 compared with V. ^##^, *p*<0.01 compared with V in the presence of BAY 11–7082. (E) Whole cell extracts, cytoplasmic fractions, and nuclear fractions were harvested from cultured cells and subjected to Western blot analysis with specific Abs as indicated. Abs for β-actin and lamin B1 were used for normalization of cytoplasmic and nuclear extracts, respectively. Numbers between the panels are ratios of the intensity of NFAT2 to β-actin (total and cytosolic) or lamin B1 (nucleus). (F) BMMs were cultured with M-CSF and RANKL for 42 h and then treated with cilostazol (30 μM) or sp-cAMP (10 μM) for 6 h. Whole cell lysates were immunoprecipitated with anti-NFAT2 and subjected to Western blot analysis with a phosphorylated PKA substrate-specific Ab. Similar results were obtained in three independent experiments.

RANKL induces RANK-proximal signals consisting of key transcription factors that converge to promote expression of OC-specific genes [13, 17]. We evaluated by EMSA whether cilostazol influences RANKL-induced NF-κB activation. BMM was stimulated by RANKL to generate NF-κB-DNA binding activity (lane 2), and cilostazol attenuated its binding in a dose-dependent pattern (lane 3 and 4) (**Fig 4A**). Competition assays using an excess of unlabeled probe was performed to show the specificity of the binding activity (lane 5). NF-Y DNA binding activity was measured as a loading control. A pharmacological inhibitor of Iκβα phosphorylation, BAY11-7082, attenuated the inhibitory effect of cilostazol on OC formation and ROS levels (**Fig 4B and 4C**), supporting the view that decreased NF-κB activation by cilostazol is partly responsible for reducing OC formation and sustaining levels of ROS. We next examined the effect of cilostazol on the expression of NFAT2, a master switch for osteoclastogenesis [13]. RANKL stimulation for 48 h in BMM increased NFAT2 mRNA, and cilostazol inhibited it (**Fig 4D**). We determined the expression and cellular location of NFAT2 protein during osteoclastogenesis induced by RANKL. As shown in **Fig 4E** (upper panel), we observed a decreased level of total NFAT2 protein in cilostazol-treated cells. RANKL greatly elevated recruitment of NFAT2 to the nuclear region of OCs, an effect again opposed by cilostazol, whereas it had not much effect on cytosolic NFAT2 (**Fig 4E**, middle and bottom panels). These observations suggest that the increase of cAMP due to cilostazol may interfere with an NFAT2 autoamplification loop by preventing the nuclear translocation of NFAT2, resulting in reduced RANKL-induced NFAT2 expression. Nuclear localization of NFAT2 is affected by phosphorylation. We therefore examined whether PKA is responsible for the reduced nuclear translocation of NFAT2 in response to cilostazol by assessing the phosphorylation status of NFAT2. Lysates of RANKL-stimulated OC were immunoprecipitated with NFAT2 and immunoblotted with antibody specific for phosphorylated PKA substrates. As shown in **Fig 4F**, cilostazol enhanced NFAT2 phosphorylation of the PKA phosphorylation sites. As a positive control, sp-cAMP also increased NFAT2 phosphorylation, whereas RANKL alone decreased it.

## Discussion

In our present studies we have shown that cilostazol induces the elevation of intracellular cAMP in OC and protects from bone loss and oxidative stress induced by OVX in mice, suggesting a protective role of cAMP in maintaining bone mass. Cilostazol has been used as a vasodilating antiplatelet drug as a potent cyclic nucleotide PDE3 inhibitor, leading to elevation of cAMP [18], but recent findings have revealed that it also improves insulin resistance [19] and decreases atherosclerosis via an anti-inflammatory activity [7], implying that increased cAMP may affect a variety of metabolic regulatory processes. In addition, cAMP improves learning and memory in mice [20]. The beneficial effect of cAMP on bone has been suggested by the findings in several systems [2, 21–24]. Resveratrol which acts as a PDE inhibitor to activate cAMP signaling improves age-related metabolic syndromes [21] while it attenuates bone loss in vivo [22, 23]. Most cannabinoid receptor antagonists activate cAMP production and mice with type 1 cannabinoid receptor deficiency exhibit increased peak bone mass due to reduced bone resorption [24]. Conversely, the detrimental effect of increased FSH induced by OVX on bone mass is also associated with decreased cAMP in OC [2]. It is plausible that increased cAMP improves undesirable effects due to elevated FSH upon loss of ovarian function. On the contrary, it is possible that elevation of cAMP at old ages results in decreased bone formation and increased adipogenesis, leading to age-related bone loss [24]. Additionally clinical studies showed that side effect of cilostazol should be considered in patients suffering atrial failure, diabetes mellitus, hepatic impairment and use with concomitant drugs that increase bleeding risk [25]. Taken together, elevation of cAMP in a global scale by cilostazol could be beneficial to alleviate bone loss for postmenopausal women who are also at high risk of metabolic disease [26], atherosclerosis [27] or cognitive impairment due to Alzheimer disease [28], compared to bone targeting drugs.

We showed that cilostazol decreased the number of OC in vivo, ex vivo cultured OC, and serum CTX-1, all of which were elevated by OVX. In agreement with the in vivo results, RANKL-induced OC formation and bone resorption assessed on dentine slices were attenuated by cilostazol in vitro. In vivo cilostazol also showed a tendency to improve trabecular microarchitecture in sham mice, although it was not statistically significant except Tb. N. Consistent with these findings, ex vivo OC from whole bone marrow exhibited a similar tendency to those from BMM, excluding a contribution of stromal/osteoblast cells to the protective activity of cilostazol. In addition, cilostazol did not affect osteoblast differentiation from bone marrow stromal cells (data not shown).

Cyclic AMP signaling is highly complex and has different effects depending on the direct and indirect effectors activated by cAMP which binds to 2 types of effector: PKA and Epac1 [16, 29]. Our results indicated that elevation of cAMP due to cilostazol increased PKA activity and inhibits NFAT2 nuclear translocation, and that activation of PKA by cilostazol was responsible, at least in part, for limiting osteoclastogenesis. An inhibitory effect of cAMP on NFAT2 has been reported in OC and T cells [15, 30]. RANKL induces adenylate cyclase, resulting in increased cAMP [15]. Elevated cAMP in naïve T regulatory cells exerts a suppressive effect via inhibition of NFAT transcription [30]. Another effector of cAMP, Epac1 is reported to have the opposite effect on NFAT in cardiac cells. Epac activates the Ca^2+^ sensitive phosphatase, calcineurin, and its primary downstream effector, NFAT, and induces cardiomyocyte hypertrophy [31], suggesting a different mechanism for the effect of Epac1 on OC formation. We also showed that cilostazol decreased NF-κB activation, another key transcription factor for OC, by demonstrating attenuated NF-κB-DNA binding activity upon RANKL stimulation. Inhibition of NF-κB by BAY11-7082, an inhibitor of IκBα phosphorylation reduced the inhibitory effect of cilostazol on OC formation as well as ROS levels, suggesting that decreased NF-κB activation is responsible for the inhibitory effect of cilostazol on OC formation. For decades the anti-inflammatory effect of cAMP has been known to be partly due to the interference with the function of pro-inflammatory NF-κB, although its effector, PKA, promotes NF-κB activity [29], implying that another effector may act to decrease NF-κB activation. Adiponectin which mimics cAMP action in renal tubular cells results in decreased NF-κB activation, having the same effect as the specific Epac activator [32]. It is also possible that Epac may be involved in attenuated NF-κB activation upon cilostazol treatment in OC.

RANKL-induced ROS generation is essential for osteoclastogenesis [33, 34]. We showed that cilostazol, sp-cAMP, and forskolin considerably reduced the sustained level of ROS in OC, showing that cAMP is responsible for decreasing ROS. Our findings further imply that both PKA and Epac1 are necessary for cilostazol to decrease ROS. Since ROS is associated with NFAT2 activation in OC [33], the reduced expression and nuclear localization of NFAT2 can be explained by decreased ROS levels in response to cilostazol.

Taken together, our results demonstrate that cilostazol attenuates the bone loss induced by elimination of ovarian function in mice. It inhibits OC formation as well as reduces levels of ROS via PKA and Epac1, leading to impaired activation of NF-κB and NFAT2. Direct phosphorylation of NFAT2 by activated PKA can explain its decreased nuclear localization, which disrupts autoamplification, leading to reduced osteoclastogenesis. These results suggest that cilostazol may be therapeutically beneficial in protecting from bone loss as well as oxidative stress caused by loss of ovarian function.

## Materials and Methods

### Ethics statement

All mice were handled in accordance with the guidelines of the Institutional Animal Care and Use Committee (IACUC) of the Immunomodulation Research Center (IRC), University of Ulsan. IACUC of IRC approved all animal procedures (approval ID for this study: # UOU2012-001).

### Animals and Study Design

Sham operation (n = 11), or OVX (n = 14) was performed for 6 week-old C57BL/6J mice under anesthesia using a mixture of Zoletil and Rompun. Mice were accommodated in the specific pathogen-free animal facility of IRC. Cilostazol, a generous gift from Otsuka Pharmaceutical Co., Ltd (Tokushima, Japan) was injected intraperitoneally at a dose of 0.5 mg/kg/body weight/day (OVX, n = 7; sham, n = 6) or vehicle (OVX, n = 7; sham, n = 5), once a day for 8 weeks after surgery. Treated dose of cilostazol was calculated on the basis of safe levels in animal studies [35]. Mice were sacrificed by CO_2_ asphyxiation. Femurs were analyzed by scanning in a high-resolution μCT imaging system using a SkyScan 1072 System (SkyScan, Kontich, Belgium) set to a 6.9 μm effective detector pixel size and a threshold of 77–255 mg/cc. Trabecular bone was assessed in a 1.5 mm region 0.2 mm below the distal growth plate of the femur. Three D analyses were performed with CT volume software (ver. 1.11; SkyScan) with a total of 250–300 tomographic slices. *In vivo* marker of bone resorption was determined by serum CTX-1 by RatLaps EIA according to the manufacturer’s directions (Immunodiagnostic Systems Inc., Woburn, MA). Serum osteocalcin was measured with an osteocalcin EIA kit (Biomedical Technologies Inc., Stoughton, MA, USA) and alkaline phosphatase (ALP) by a colorimetric kinetic assay (BioAssay Systems, Hayward, CA, USA). Serum H_2_O_2_ was evaluated with an Amplex Red hydrogen peroxide/peroxidase assay kit (Invitrogen, Carlsbad, CA).

### OC formation

Bone marrow cells were isolated from C57BL/6J mice as described [36]. Cells from sham and OVX mice were obtained 2 weeks after surgery, since 2 week has been used to investigate the acute phase of OVX-induced bone loss including ex vivo OC formation [37]. Femora and tibiae were removed aseptically and dissected free of adherent soft tissue. The marrow cavity was washed with α-MEM from one end of the bone using a sterile 21-gauge needle after dissecting the bone ends, and a single cell suspension was prepared with a Pasteur pipette. The resulting bone marrow suspension was washed twice, and incubated on plates along with M-CSF (20 ng/ml) for 16 h. Floating cells were harvested and loaded on a Ficoll-hypaque gradient and the cells at the interface were harvested. Two more days of incubation resulted in large populations of adherent monocyte/macrophage-like cells that were seated on the bottom of the culture plates, as described before [36]. Floating cells were discarded by washing the dishes with phosphate-buffered saline (PBS), and the adherent cells (bone marrow-derived macrophages (BMM)) were collected, and seeded on plates. The adherent cells were analyzed with a FACSCalibur flow cytometer (Becton Dickinson, Franklin Lakes, NJ) and found to be negative for CD3 and CD45R, and positive for CD11b. The lack of growth without M-CSF confirmed the absence of contaminating stromal cells. M-CSF and RANKL (40 ng/ml) were added to the cells, and the incubated medium was replaced on day 3. After incubation for the indicated times, the cells were fixed in 10% formalin for 10 min, and stained for TRAP as described [36]. OC numbers were evaluated by an experienced observer who was blinded to each treatment for quantification of TRAP-positive multinucleated cells (MNC) (three or more nuclei)/ each well using an eye piece graticule at a magnification of Χ100. The cultures with and without RANKL in the presence of M-CSF were used as positive and negative control, respectively. The BMM were transfected with small interfering RNA (siRNA) against Epac1 (siEpac1) (sc-41701; Santa Cruz Biotechnology), protein kinase A catalytic subunit α (siPKAα) (sc-36241), p47^phox^ (sip47^phox^) (sc-36157) or scrambled siRNA (scRNA) (Santa Cruz, Santa Cruz, CA) using Lipofectamine RNAiMAX (Invitrogen), and further analyzed. Lipofectamine which was diluted in α-MEM was mixed with an equal volume of α-MEM containing the siRNA. After 20 min of incubation, 100 μl of RNAiMAX/siRNA was added to the cells, reaching a final volume of 700 μl. After 8 h incubation, the cells were replated in serum-containing medium, cultured for another 2 d, and mRNA levels were analyzed qPCR. OC were further characterized by assessing their ability to form pits on dentine slices [38]. Mature OC cells were generated by incubation with M-CSF and RANKL for 5 d. Then, after treatment with EDTA, the cells were harvested [39]. Mature OC were seeded on dentine slices and incubated for 1 d with M-CSF and RANKL. Mature OC with and without RANKL in the presence of M-CSF were used as positive and negative control, respectively. The slices were cleaned by ultrasonication in 1M NH_4_OH to remove adherent cells and stained with Mayer’s hematoxylin (Sigma) to visualize resorption pits.

### RNA Isolation and Quantitative Polymerase Chain Reactions (qPCR)

Total RNA was reverse-transcribed with oligo-dT and Superscript I (Invitrogen). qPCR was carried out using SYBR Green 1 Taq polymerase (Qiagen, Hilden, Germany) and appropriate primers on a DNA Engine Opticon Continuous Fluorescence Detection System (MJ Research Inc., Waltham, MA). The specificity of each primer pair was confirmed by melting curve analysis and agarose-gel electrophoresis. The housekeeping GAPDH gene was amplified in parallel with the genes of interest. Relative copy numbers compared to GAPDH were calculated using 2^-∆∆Ct^. The primer sequences used were as follows: 5’-gaccaccttggcaatgtctctg-3’ and 5’-tggctgaggaagtcatctgagttg-3’ (TRAP); 5’-aataacatgcgagccatcatc-3’ and 5’-tcaccctggtgttcttcctc-3’ (NFAT2); 5’-tgaggcttctcttggtgtccatac-3’ and 5’aaagggtgtcattactgcggg-3’ (cathepsin K); 5’- ctgctcctagtgagcccaac-3’ and 5’-cagcaatcgacaaggagtga-3’ (calcitonin receptor); 5’-agacgtggtttaggaatgcagctc-3’ and 5’-tcctccatgaacaaacagttccaa-3’ (DC-STAMP); 5’-ttcagttgctatccaggactcgga-3’ and 5’-gcatgtcatgtaggtgagaaatgtgctca-3’ (ATP6v0d2); 5’-gcttcctccagaaactctca-3’ and 5’-aacgctgccatcacttctct-3’ (Epac1); 5’-gcaaaggctacaacaaggc-3’ and 5’-atggcaatccagtcagtcg-3’ (PKAα); 5’-gatgttccccattgaggccg-3’ and 5’-gtttcaggtcatcaggccgc-3’ (p47^phox^); 5’-acccagaagactgtggatgg-3’ and 5’-cacattgggggtaggaacac-3’ (GAPDH).

### The enzyme-linked immunoabsorbent assay (ELISA) for cAMP

The cAMP ELISA was performed according to the manufacturer’s instructions (Oxford Biomedical Research, Oxford, MI). Samples were processed using the acetylation protocol. Trimethylbenzidine was used as a substrate for horse radish peroxidase (HRP) and absorbance was measured at 450 nm.

### EMSA

Biotinylated double-stranded oligonucleotides were synthesized by Bioneer Co. (Korea): NF-κB, 5'-agttgaggggactttcccaggc-3'; NF-Y, 5’-agaccgtacgtgattggttaatctctt- 3’. Nuclear extracts were prepared from BMM stimulated with RANKL (40ng/ml) using NE-PER nuclear and cytoplasmic extraction reagents (Pierce, Rockford, IL, USA). Binding reactions were carried out for 20 min at room temperature in the presence of 50 ng/ml poly(dI-dC), 0.05% Nonidet P-40, 5 mM MgCl_2_, 10 mM EDTA, and 2.5% glycerol in 1x binding buffer using 20 fM of biotin-end-labeled target DNA and 3 μg of nuclear extract (LightShift Chemiluminescent EMSA kit; Pierce). Samples were loaded onto native 6% polyacrylamide gels pre-electrophoresed for 60 min in 0.5x Tris borate/EDTA and run at 100 V before being transferred onto a positively charged nylon membrane (Hybond-N+) in 0.5 X Tris borate/EDTA at 100 V for 30 min. Transferred DNAs were cross-linked to the membrane at 10 mJ/cm^2^ and detected using HRP-conjugated streptavidin.

### Fractionation and Western Blot Analysis

Cultured cells were harvested after washing with ice-cold PBS and lysed in extraction buffer (50 mM Trsi-HCl, pH 8.0, 150 mM NaCl, 1 mM EDTA, 0.5% Nonidet P-40, 0.01% protease inhibitor mixture). Cells were fractionated using Nuclear and Cytoplasmic Extraction reagents (Pierce). Cytoplasmic and nuclear extracts were subjected to SDS-PAGE and transferred onto nitrocellulose. Membranes were blocked for 1h with skim milk in Tris-buffered saline containing 0.1% Tween 20% and incubated overnight at 4°C with anti-NFAT2 (0.2 μg/ml), anti-lamin B1 (0.1 μg/ml; Santa Cruz) and anti-β-actin (2.2 μg/ml; Sigma). Membranes were washed, incubated for 1h with HRP-conjugated secondary Abs (BD Biosciences), and developed using chemiluminescent substrates.

### Detection of Intracellular Reactive Oxygen Species (ROS)

Intracellular ROS were detected with the fluorescent probe 2’,7’-dichlorofluorescein diacetate (H_2_DCFDA) (Molecular Probes). BMMs were loaded with H_2_DCFDA, and incubated at 37°C for 30 min. Intracellular ROS were measured with a FACSCalibur flow cytometer.

### Statistical Analysis

Values are expressed as means ± SEM. Pairs of groups were compared by Student’s *t*-test and multiple groups by one-way ANOVA followed by Bonferroni post tests. A *P* value of less than 0.05 was considered statistically significant.

## Supporting Information

S1 Fig Cilostazol does not affect cell viability. Cell viability was measured by MTT assay. BMMs from SHAM (open bar) and OVX (obscure bar) mice were incubated without cilostazol (V) or with cilostazol (15, 25 μM) in the presence of M-CSF (20 ng/ml) and RANKL (40 ng/ml). After 3d, cells were washed and incubated with MTT (3-(4,5-Dimethylthiazol-2-yl)-2,5-Diphenyltetrazolium Bromide) for 3h and lysed in 50% dimethylformamide. Absorbance was determined at 595 nm with a microplate reader. ns, no significant difference between each dose of cilostazol treated cells and V. Similar results were obtained in 3 independent experiments.
(TIF)

S2 Fig Cilostazol does not affect OC survival. Survival of osteoclasts was measured by counting TRAP+ MNCs. BMMs were incubated with M-CSF (20 ng/ml) and RANKL (40 ng/ml) for 3d to get mature OCs. Following washed by PBS twice, OCs were refreshed by new culture media, and cilostazol were treated as different dose (10, 20, 30 μM) or not (V) in the presence of M-CSF (20 ng/ml) and RANKL (40 ng/ml). After 12hrs, survived OCs were identified by TRAP-positive MNCs which containing nuclei more than 3. ns, no significant difference between each dose of cilostazol treated cells and V. Similar results were obtained in 3 independent experiments.
(TIF)
