# Supplementary figures and images for: Cilostazol Attenuates Ovariectomy-Induced Bone Loss by Inhibiting Osteoclastogenesis
Source: PLoS One. 2015 May 18;10(5):e0124869. doi: 10.1371/journal.pone.0124869 (PMC4436362; doi:10.1371/journal.pone.0124869)

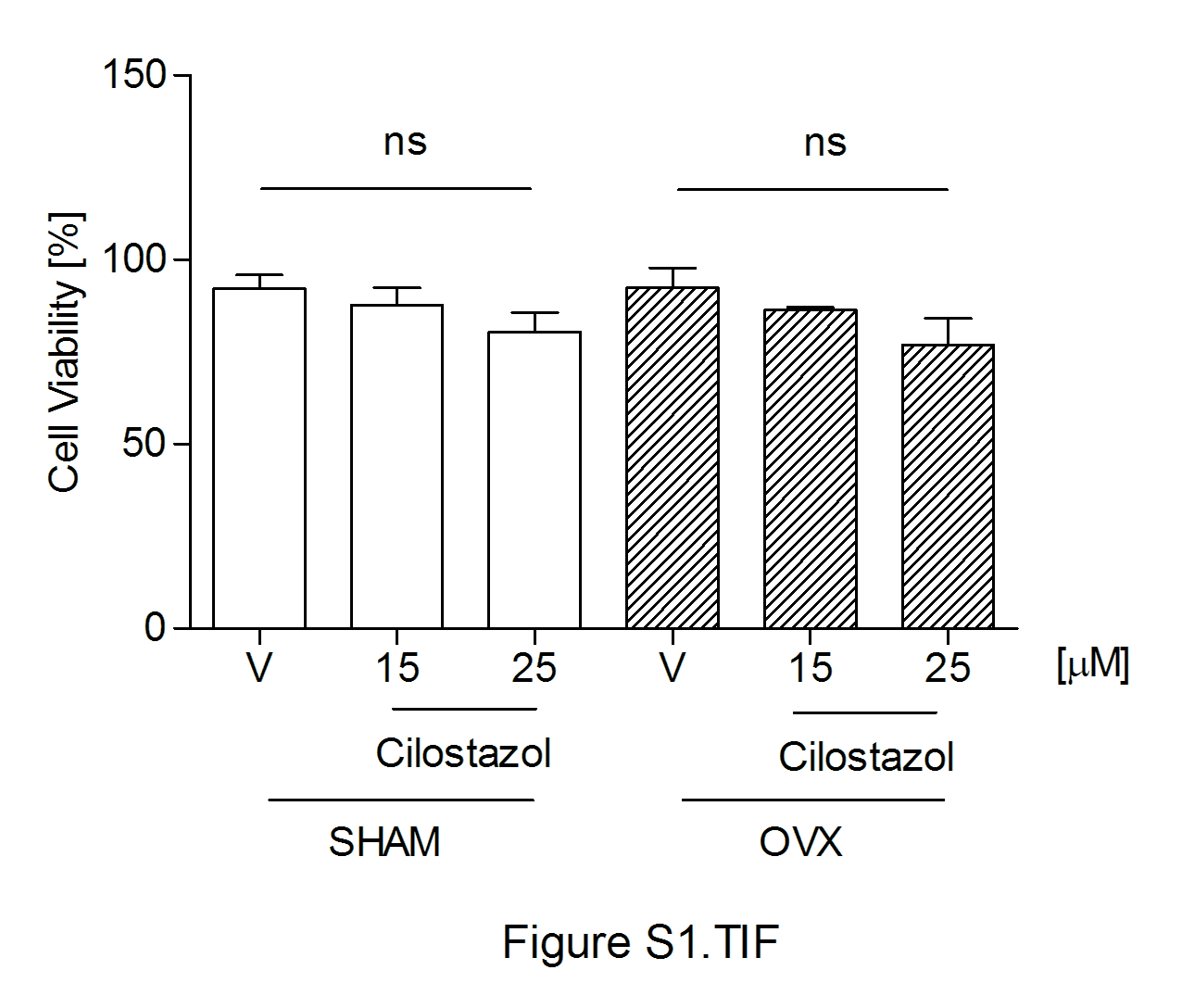

Supplement: S1 Fig — Cell viability was measured by MTT assay. BMMs from SHAM (open bar) and OVX (obscure bar) mice were incubated without cilostazol (V) or with cilostazol (15, 25 μM) in the presence of M-CSF (20 ng/ml) and RANKL (40 ng/ml). After 3d, cells were washed and incubated with MTT (3-(4,5-Dimethylthiazol-2-yl)-2,5-Diphenyltetrazolium Bromide) for 3h and lysed in 50% dimethylformamide. Absorbance was determined at 595 nm with a microplate reader. ns, no significant difference between each dose of cilostazol treated cells and V. Similar results were obtained in 3 independent experiments. (TIF) [file pone.0124869.s001.tif]

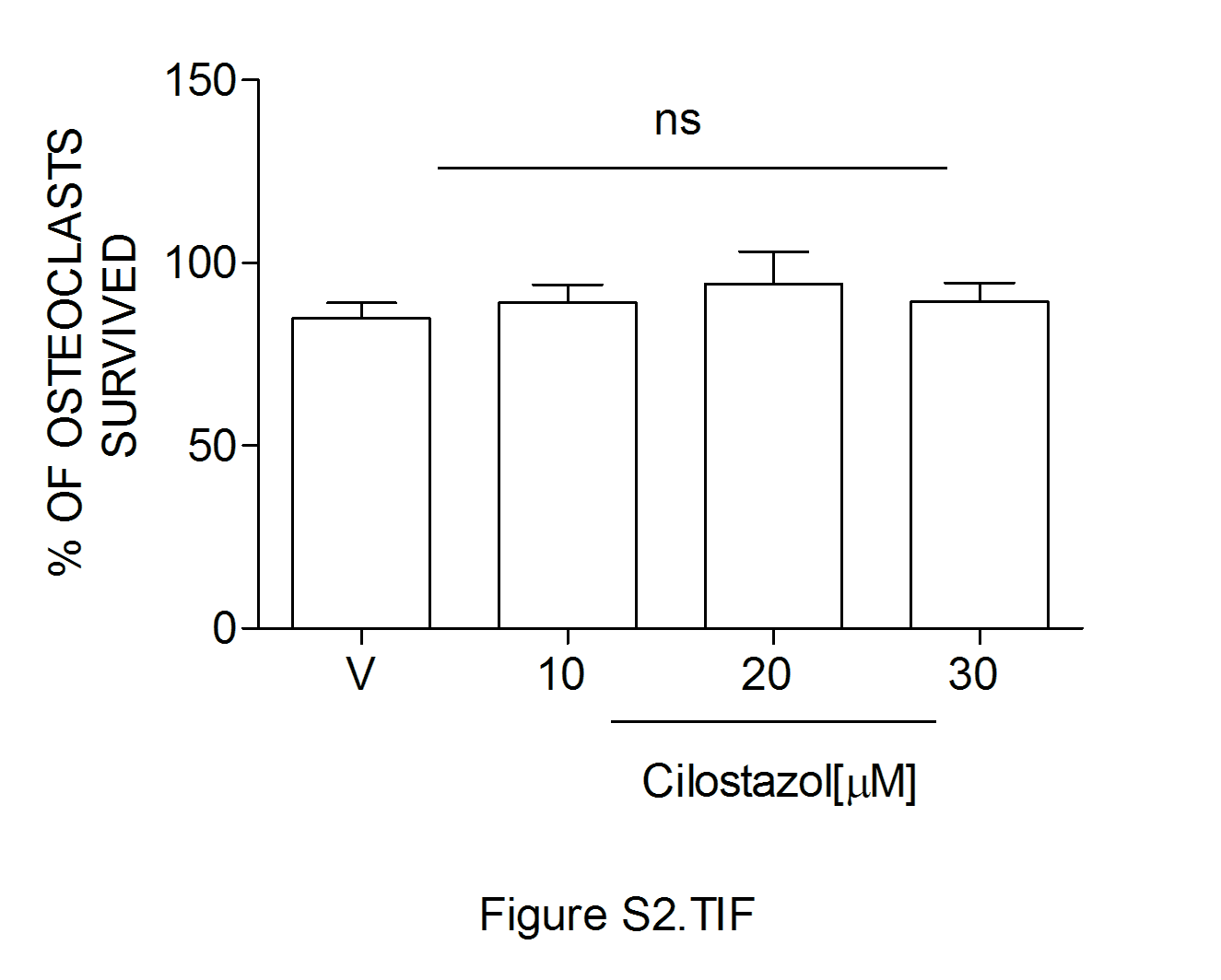

Supplement: S2 Fig — Survival of osteoclasts was measured by counting TRAP+ MNCs. BMMs were incubated with M-CSF (20 ng/ml) and RANKL (40 ng/ml) for 3d to get mature OCs. Following washed by PBS twice, OCs were refreshed by new culture media, and cilostazol were treated as different dose (10, 20, 30 μM) or not (V) in the presence of M-CSF (20 ng/ml) and RANKL (40 ng/ml). After 12hrs, survived OCs were identified by TRAP-positive MNCs which containing nuclei more than 3. ns, no significant difference between each dose of cilostazol treated cells and V. Similar results were obtained in 3 independent experiments. (TIF) [file pone.0124869.s002.tif]
